# Causes of Deaths in Children under-Five Years Old at a Tertiary Hospital in Limpopo Province of South Africa

**DOI:** 10.5539/gjhs.v5n3p95

**Published:** 2013-02-15

**Authors:** Sam Thembelihle Ntuli, Ntambwe Malangu, Marianne Alberts

**Affiliations:** 1 Department of Public Health Medicine, University of Limpopo, Polokwane Campus, Gauteng, South Africa; 2 Department of Epidemiology and Biostatistics, University of Limpopo, Medunsa Campus, Gauteng, South Africa; 3 Department of Medical Sciences, University of Limpopo, Polokwane, South Africa

**Keywords:** causes of death, neonates, mortality, Limpopo Province

## Abstract

**Objective:**

Accurate and timely information on the causes of child deaths is essential in guiding efforts to improve child survival, by providing data from which health profiles can be constructed and relevant health policies formulated. The purpose of this study was to identify causes of death in children younger than 5 years-old in a tertiary hospital in South Africa.

**Methods:**

Death certificates from the Pietersburg/Mankweng hospital complex, for the period of January 1, 2008 through December 31, 2010, were obtained for all patients younger than 5 years and were retrospectively reviewed. Data were collected using a data collection form designed for the study. Information abstracted included: date of death, age, sex, and cause of death.

**Results:**

A total of 1266 deaths were recorded, the sex ratio was 1.26 boys per girl. About 611 (48%) of deaths were listed as neonatal deaths (0-28 days), 387 (31%) were listed as infant deaths (29 days-11 months), and 268 (21%) as children’s death (1-4 years). For neonates the leading causes of death were: prematurity/low birth weight, birth asphyxia and pneumonia. For the infant death group, the leading causes of death were pneumonia, diarrhea, and HIV/AIDS; and in the children’s group, the leading causes were injuries, diarrhea and pneumonia. There was no statistical significant difference in the proportions of causes of death based on the sex of children.

**Conclusion:**

The top 10 leading causes of death in children under-5 years old treated at Pietersburg/Mankweng Hospital Complex were in descending order: prematurity/low birth weight, pneumonia, diarrheal diseases, birth asphyxia, and severe malnutrition, HIV/AIDS, hydrocephalus, unintentional injuries, meningitis and other infections. These ten conditions represent 73.9% of causes of death at this facility. A mix of multi-faceted interventions is needed to address these causes of death in children.

## 1. Introduction

Accurate and timely information on the causes of child deaths is essential in guiding efforts to improve child survival, by providing data from which health profiles can be constructed and relevant health policies formulated (Bradshaw et al., 2003). Such data are not only crucial for monitoring the causal factors that lead people to die, but also to establish the patterns of medical causes of death. In doing so, such data are helpful for targeting where, when, and how public health resources should be channeled to address these issues and avoid untimely deaths.

In the case of South Africa, data from the Death Notification Forms (DNF’s) as compiled by Statistics South Africa conform to the World Health Organization (WHO) guidelines; they are good sources of information on death and its causes. Moreover, it is established that about 48.6% of deaths in South Africa occur within a healthcare facility (Statistics South Africa [Stats SA], 2011a). Hence, institutional death registers are valuable repository of data that could be analyzed and used for local decision-making purposes. This is important because in order to achieve the Millennium Goal 4, that is to reduce child mortality, a concerted effort is needed from local to national institutions.

Nationally, the most recent data on child mortality come from the 2007 Community Survey which reported that under-five mortality rate was 104 per 1000 live births which is higher than the international target of 20 per 1000 live births (Stats, 2011b). In order to assist local healthcare teams in knowing which health conditions to focus on, there is a need to identify the causes of death prevalent in their settings. Therefore, a retrospective descriptive study was undertaken to establish the common causes of death of children under-five year old at a tertiary hospital of the Limpopo Province.

## 2. Material and Methods

This was a retrospective descriptive study based on the review of records at Pietersburg/Mankweng Hospital Complex (PMHC), the only tertiary referral hospital in Limpopo Province of South Africa. It is a teaching hospital for the University of Limpopo (Medunsa Campus) that mainly serves rural communities. The neonatal unit at PMHC has a maximum capacity of 54 beds and a small Neonatal Intensive Care Unit with 9 beds. The unit has a total admission of 800 patients per annum. The data for this study was collected for a period of 36-months from January 1, 2008 to December 31, 2010. Data were collected using a data collection form designed for the study. For the purpose of this study, a neonatal death was defined as a death occurring on or before 28 days of life, infant death as a death occurring between the 29^th^ day of life and the 11^th^ month of life, and children’s death as a death occurring between the 1^st^ year of life and the 4^th^ year of life.

The hospital death register was used as the starting point to collate the list of deaths that occurred during the study period. Then the relevant death notification forms were retrieved. In order to increase the reliability, the patients’ register and the files were also consulted. Using the register, patient files and the death notification forms, the following data were collected: date of death, gender and age of the deceased, and the cause of death. Although the death notification form contains the information about which method was used to ascertain the cause of death, it is well established that in South Africa about 60% of deaths are ascertained from the opinions of medical doctors and nurses (Stats, 2011a).

The recorded causes of death were classified the according to the International Classification of Disease version 10 (ICD-10). Stillbirths were excluded from the study. The statistical software, STATA version 9.0 (StataCorp; College Station, Texas) was used for the data analysis. Comparison was performed using chi-square test for categorical variables. The p-values of less and/or equal to 0.05 were considered statistically significant. Ethical clearance was granted by the Medunsa Campus Research Ethics Committee of the University of Limpopo.

## 3. Results

A total of 1266 deaths in children under-5 years old were reported during the study period. Of this group, 55.8% (706/1266) were males; with a male to female ratio of about 1.26 boys per girl. Neonatal deaths accounted for about half of the deaths recorded (48.3%); while infant and children deaths accounted for 31% (387/1266) and 21% (268/1266) of total deaths respectively. There was no statistical significant difference (p=0.884) in the proportions of causes of death based on the sex of children (Table 1).

**Table 1:** Deaths by age and sex among under-five year's old children at PMHC 2008-2010

Age category	Male		Female		Total	

	N	%	N	%	N	%
Neonatal (0-28 days)	345	48.9	266	47.5	611	48.3
Infant (29 days-11mths)	214	30.3	173	30.9	387	30.6
Children (1-4 years)	147	20.8	121	21.6	268	21.2
**Total**	**706**	**100.0**	**560**	**100.0**	**1266.0**	**100.0**

With regard to the causes of deaths, there were noticeable differences among the three groupings. Among neonates, just over half of the deaths were due to prematurity/low birth weight; the other most prevalent causes in this group were birth asphyxia and pneumonia. In the infant group, pneumonia was the most prevalent cause of death (31.8%) followed by diarrhea and HIV/AIDS. The three causes constituted over a 50% of all causes recorded in this group. In children, unintentional injuries contributed 14.2% (38/268) of deaths. Together with diarrhea, pneumonia, meningitis and severe malnutrition, they constituted over 50% of all deaths recorded in this group (Table 2). Of the injuries recorded, 52.6% (20/38) of them were burns, 31.6% (12/38) were poisoning, while 15.8% (6/38) were road traffic accidents. During the period of the study, there were no changes in the pattern of deaths along the years in relation to the total death by age group and cause of death (data not shown).

**Table 2:** Causes of death in under-five years old children at PMHC 2008-2010

Neonates (<29 days)			Infant (29 days -11 months)			Children (1 - 4 years)		
Causes	N	%	Causes	N	%	Causes	N	%
Prematurity/low birth weight	319	52.2	Pneumonia	123	31.8	Unintentional injuries	38	14.2
Birth asphyxia	79	12.9	Diarrhoea	51	13.2	Diarrhoea	34	12.7
Pneumonia	30	4.9	Undetermined/unknown	31	8.0	Pneumonia	29	10.8
Undetermined/unknown	29	4.7	HIV/AIDS	25	6.5	Severe malnutrition	26	9.7
Congenital anomalies	27	4.4	Hydrocephalus	24	6.2	Meningitis	21	7.8
Neonatal Sepsis	25	4.1	Severe malnutrition	24	6.2	Other infection	18	6.7
Meconium aspiration	13	2.1	Prematurity/low birth weight	20	5.2	Hydrocephalus	16	6.0
Renal failure	11	1.8	Meningitis	14	3.6	Undetermined/unknown	16	6.0
HIV/AIDS	9	1.5	Bowel obstruction	10	2.6	HIV/AIDS	12	4.5
Respiratory failure	7	1.1	Other infection	10	2.6	Malignancies	12	4.5
Diarrhoea	7	1.1	Sepsis	7	1.8	Tuberculosis	11	4.1
Other infection	6	1.0	Respiratory failure	4	1.0	Bowel obstruction	5	1.9
Miscellaneous	49	8.0	Miscellaneous	44	11.4	Miscellaneous	30	11.2
**Total**	**611**	**100.0**	**Total**	**387**	**100.0**	**Total**	**268**	**100.0**

Overall, as shown in Table 3, the top 10 leading causes of death in children under-5 years were in descending order: prematurity/low birth weight, pneumonia, diarrheal diseases, birth asphyxia, and severe malnutrition, HIV/AIDS, hydrocephalus, unintentional injuries, meningitis and other infections. These ten conditions represent 73.9% of causes of death at the study site.

**Table 3:** The leading causes of deaths in under-five year's old children at PMHC 2008-2010

Causes	N	%
Prematurity/low birth weight	339	26.8
Pneumonia	182	14.4
Diarrhea	92	7.3
Birth asphyxia	79	6.2
Severe malnutrition	50	3.9
HIV/AIDS	46	3.6
Hydrocephalus	40	3.2
Unintentional injuries	38	3.0
Other infection	34	2.7
Meningitis	35	2.8
Sepsis	32	2.5
Congenital abnormalities	27	2.1
Bowel obstruction	15	1.2
Meconium aspiration	13	1.0
Malignancies	12	0.9
Renal Failure	11	0.9
Tuberculosis	11	0.9
Respiratory failure	11	0.9
Miscellaneous	123	9.7
Undetermined/unknown	76	6.0
**Total**	**1266**	**100**

## 4. Discussion

This study shows that several health conditions are the causes of death in under-five year’s old children. As discussed below the distribution of these causes varies with the ages of the children. The male to female ratio reported in this study is slightly higher than the national figure of 1.12 boys per girl (Nannan et al., 2012). It is worth noting that 48% of all deaths among under-5 years old occurred during the neonatal period. This finding concurs with the reports from a study conducted in KwaZulu-Natal Province that reported that more than 48% of deaths among children under-5 years old occurred in infants aged 29 days to 11 months old (Stats, 2009; Stephen et al., 2009; Garrib et al., 2006). In contrast, a population-based study in rural area of Limpopo Province of South Africa reported that 46% of deaths among children under-5 years old were among those aged 1 to 4 years old (Agincourt health and socio-demographic surveillance site [AHDSS], 2002). Another population-based study in Maputo Province of Mozambique indicated that 41% of under-five deaths were among children aged 1-4 years (Sacarlal et al., 2009). These findings are again in contrast to reports by Stephen and Patrick (2008) that 59% of deaths were among infants. It seems that the aggregation of death data at provincial and national levels masks important local or institutional disparities.

With regard to the causes of death, this study found that the most common causes reported in neonates were prematurity or low birth weight, birth asphyxia, upper respiratory infections such pneumonia, and neonatal sepsis. These findings concur with reports by other investigators (Garrib et al., 2006; Liu et al.,2011; Mmbaga et al., 2012; Nannan et al., 2012; Pattinson, 2009; Rashid et al., 2010; Roy et al.,2008; Stephen et al., 2009). Given the fact that over half of deaths were due to prematurity and circumstances surrounding the birth such as asphyxia and neonatal sepsis, there is a need for special attention to be paid to environmental and socioeconomic factors that affect prematurity as well as working conditions that may led the onset of sepsis in neonates.

In infants, this study shows that upper respiratory infections such as pneumonia, HIV/AIDS and diarrheal diseases constituted over half of causes of death. These findings concur with reports by Garrib and co-workers (2006) as well as Nannan and collaborators (2012). Given the infectious nature of these three dominant causes of death, it points out to the need for timeous diagnosis and appropriate treatment to be given. It is important that local Pharmacy and Therapeutics Committees be involved in ensuring that appropriate care and treatment protocols are adhered to and that the relevant medicines are available to treat these infections.

In children aged 1-4 years, unintentional injuries together with diarrhea, pneumonia, meningitis and severe malnutrition constituted over 50% of deaths. This finding is consistent with reports by previous investigators who stated that infections such as HIV/AIDS, diarrheal diseases, upper respiratory infections such as pneumonia as well as malnutrition were the most common causes of death (Nannan et al., 2012; Stephen et al., 2009; Garrib et al., 2006). However, in this rural setting, this study shows that unintentional injuries are the leading causes of death in this group of children. This finding is consistent with previous findings by Malangu (2005) that acute poisoning was responsible for 17% of admissions to wards in a rural hospital of Limpopo Province. Moreover, the proportion of deaths due to injuries as reported in this study is higher than the national average of 12.5% of deaths due to unnatural causes in this group (Stats, 2011a). Because deaths through injuries are avoidable, a public health education campaign is needed to raise the communities’ awareness on prevention strategies.

Taken together the above findings suggest that congenital conditions, infections, malnutrition and unintentional injuries contribute significantly to the deaths of under-five year’s old children. It is clear therefore that to address the Millennium Goal 4, in particular, to reach the targeted under-five mortality rate of 20 per 1000 live births, a mix of multi-faceted interventions is needed in addition to what was stated above. These interventions include the improvement in the care of and the environment for children; the support to home caregivers to children; the provision of safe and secure playgrounds; the strengthening of healthcare aspects relating to children. For instance, the majority of deaths reported in this study were due to prematurity/low birth weight. To manage neonates suffering from this condition specialised care such as intensive care units are needed together with highly trained personnel. Previous studies have reported that the establishment of neonatal intensive care units and the use of mechanical ventilation and exogenous surfactants improve the outcomes of neonates suffering from prematurity (Hack et al., 1996; Draper et al., 1999; Richardus et al., 2003; Velaphi et al., 2005). These reports suggest the need for upgrading the infrastructure of rural hospitals in order to decrease unnecessary deaths among children.

The above findings must be considered taking into account the following limitations. This study was limited to three years; a longer study period may show a different pattern of causes of death. Moreover, the study was conducted in a tertiary referral hospital which admits patients that could not be managed at lower level of care due to complications; therefore, the situation may not be probably a real reflection of causes of child mortality throughout the province. Secondly, the recorded causes of death were based on clinical assessment of the attending physicians. Because no autopsies were performed on any of the deceased children, no post-mortem records could be used to verify the correctness of the causes of deaths recorded in the register and death notification forms. Finally, this study was a retrospective hospital record review; therefore the results cannot be extrapolated or generalized to the whole province or the whole country of South Africa.

In conclusion, the top 10 leading causes of death in children under-5 years old treated at Pietersburg/Mankweng Hospital Complex were in descending order: prematurity/low birth weight, pneumonia, diarrheal diseases, birth asphyxia, and severe malnutrition, HIV/AIDS, hydrocephalus, unintentional injuries, meningitis and other infections. These ten conditions represent 73.9% of causes of death at this facility. A mix of multi-faceted interventions is needed to address these causes of death in children.
